# Reducing tobacco-associated lung cancer risk: a study protocol for a randomized clinical trial of AB-free kava

**DOI:** 10.1186/s13063-023-07081-x

**Published:** 2023-01-18

**Authors:** Chengguo Xing, John Malaty, Melissa Bou Malham, Anna Maria Abi Nehme, Breanne Freeman, Zhiguang Huo, Roberto Firpi-Morrel, Ramzi G. Salloum

**Affiliations:** 1 Department of Medicinal Chemistry, College of Pharmacy, University of Florida, Gainesville, FL USA; 2 Department of Community Health and Family Medicine, College of Medicine, University of Florida, Gainesville, FL USA; 3 Department of Health Outcomes and Biomedical Informatics, College of Medicine, University of Florida, Gainesville, FL USA; 4 Department of Biostatistics, College of Public Health and Health Professions and College of Medicine, University of Florida, Gainesville, FL USA; 5 Division of Gastroenterology, College of Medicine, University of Florida, Gainesville, FL USA

**Keywords:** Randomized controlled trial, Tobacco dependence, Smoking cessation, Kava

## Abstract

**Background:**

Tobacco use is the leading cause of many preventable diseases, resulting in premature death or disease. Given that the majority of adult who smoke want to stop, this health burden could be significantly reduced if the success rate of tobacco cessation can be improved. In addition, most adults planning to quit were interested in trying complementary approaches to facilitating tobacco cessation, which is currently lacking. Therefore, there is an unmet and urgent need for novel interventions to improve the success of tobacco cessation. If such an intervention can reduce tobacco-associated lung carcinogenesis, that will be more desirable. The goal of this project is to develop a safe and effective kava-based intervention to enable tobacco cessation and reduce lung cancer risk, which will improve the health of smokers.

**Methods:**

A randomized controlled trial will enroll 80 adults who currently smoke at least 10 cigarettes daily and randomize 1:1 into the placebo and AB-free kava arms, being exposed for 4 weeks, with a total of six visits (weeks 0, 1, 2, 4, 8, and 12) to evaluate the compliance and potential issues of AB-free kava use among the participants, explore the potential effect of the AB-free kava intervention on tobacco dependence, tobacco use, and lung carcinogenesis biomarkers. Participants will be enrolled during their primary care clinic visit.

**Discussion:**

Primary care settings play a critical role in tobacco-related disease screening, counseling, and early intervention, as the majority of adults who smoke visit their physicians annually. Building upon our promising pilot human trial results in conjunction with ample compelling lab animal results, and consistent with evidence of kava’s benefits from epidemiological data, this trial will evaluate the compliance of AB-free kava among adults who currently smoke with no intention to quit. The other exploratory aims include (1) whether AB-free kava intervention can reduce tobacco use and tobacco dependence; (2) whether AB-free kava use suppresses tobacco-induced carcinogenesis; and (3) the potential of the mechanism-based noninvasive biomarkers in precision AB-free kava intervention. The positive results from this study are expected to provide a great opportunity to effectively reduce smoking rates and tobacco-related diseases.

**Trial registration:**

ClinicalTrials.gov with the identifier: NCT05081882. Registered on October 18, 2021.

**Supplementary Information:**

The online version contains supplementary material available at 10.1186/s13063-023-07081-x.

## Background

### Background and rationale

Tobacco use is the leading cause of various preventable diseases in the USA and in Florida [1]. Nearly half a million American adults die prematurely of tobacco exposure annually, with lung cancer accounting for 29% of such deaths. Given the limited success in current lung cancer treatment and tobacco cessation, preventing lung carcinogenesis in conjunction with reducing cigarette use may be necessary to minimize lung cancer incidence among patients who are addicted to smoking. Our previous lab animal data [2–6] and pilot human trial results [7] indicate that kava is such a candidate, consistent with its epidemiological observations [8–10].

Kava is a beverage from the South Pacific Islands that helps reduce stress and improve sleep [11]. Kava had also been used to manage mild and moderate anxiety with no signs of withdraw or dependence [11]. We demonstrated that kava reduced DNA damage (3-methyl adenine, 3-mA, in urine) induced by 4-(methylnitrosamino)-1-(3-pyridyl)-1-butanone (a tobacco specific lung carcinogen, known as NNK) in lab animals and human adults who smoke to prevent lung carcinogenesis [2–7]. The mechanism is to enhance NNK detoxification—the increase in urinary total NNAL (4-(methylnitrosamino)-1-(3-pyridyl)-1-butanol) [3, 4, 7]. Our pilot trial discovered that kava also reduced tobacco use (the reduction in 24-h urinary total nicotine equivalents—TNE, which measures the sum of the major metabolites of nicotine as an quantitative indicator of tobacco exposure) and dependence (questionnaires) among adults addicted to smoking [7]. Suppressing stress-mediated protein kinase A (PKA) activation is the potential mechanism, because PKA is elevated in the brain of adults who smoke and contributes to tobacco addiction [12]. At the same time, poor quality of sleep and insomnia are known factors that contribute to the poor outcome of current pharmacological tobacco cessation interventions. Kava has been used traditionally to help improve the quality of sleep, which may also facilitate tobacco cessation.

We therefore hypothesize that AB-free kava, an IND enabled dietary supplement with improved safety and stringent quality control, safely and effectively helps adults who smoke reduce tobacco use/dependence and enhance carcinogen clearance, minimizing lung cancer risk. The goal of this trial is to characterize the compliance of AB-free kava use among adults who smoke, evaluate its potential to reduce tobacco use/dependence, and explore the reduction in lung carcinogenesis risk via a double-blind randomized, placebo controlled 4-week AB-free kava intervention trial. The potential of precision AB-free kava intervention will be explored as well. The sample size (80 participants, 40 in each arm with 32 expected to complete the trial) is based on the extent of tobacco use reduction in the pilot trial [7]. The AB-free kava dose regimen (3 x 75 mg daily) is based on its recommended use in human as a dietary supplement [11].

### Objectives

This study has two aims: (1) to document AB-free kava compliance and identify potential issues and (2) to validate AB-free kava’s benefits in reducing tobacco dependence, tobacco use, and lung cancer risk biomarkers. The primary objective of this study is to evaluate AB-free kava compliance and identify potential issues. Our secondary objectives are to examine whether AB-free kava has the potential to help facilitate tobacco cessation—tobacco cessation-related questionnaires and reduction in urinary TNE (total nicotine equivalents, which measures nicotine, cotinine, and 3-hydroxycotinine and their conjugates separately). The exploratory objective is to examine whether AB-free kava has the potential to reduce lung carcinogenesis risk induced by tobacco specific carcinogen—NNK (reduction in urinary 3-mA and increase in urinary NNAL).

### Trial design

This is a parallel group trial with a 1:1 allocation ratio and an exploratory framework. To rigorously test our hypothesis and build the solid foundation for future AB-free kava translation among adults who smoke, we will conduct a double-blind randomized placebo-controlled longitudinal trial (4-week AB-free kava intervention vs. placebo with two follow-ups at weeks 8 and 12). Briefly, adults who smoke at least 10 cigarettes daily with no intention to quit will be enrolled and randomized into two groups (those willing to quit will be referred to the state tobacco cessation program). One group will take placebo capsules and the other group will take AB-free kava capsules (one capsule each time, 3 times daily, each capsule has 75 mg kavalactone) for 4 weeks.

Safety monitoring and sample collections will be implemented at weeks 0, 1, 2, 4, 8, and 12 respectively. Two follow-ups (weeks 8 and 12) are designed to (1) explore the sustainable potential of AB-free kava and (2) capture delayed adverse effects if any, both of which are critical for participants and future development. AB-free kava regimen in the current trial remains the same as in the pilot trial [7], further supported by our recent pharmacokinetic results (the half-lives of the main ingredients in AB-free kava are no more than 3 h) [13]. The 4-week AB-free kava exposure is expected to provide sufficient data about AB-free kava’s compliance, efficacy, and safety while minimizing potential risks among the participants. At the end of week 12, all enrolled participants will be recommended to Tobacco Free Florida to maximize the chance of tobacco cessation. This study has been approved by the Institutional Review Board (IRB) under number 202101885 (IRB of Record: UF IRB-01) and is registered in ClinicalTrials.gov (NCT05081882). The protocol conforms with the SPIRIT Checklist for Trials.

## Methods

### Study setting

The trial will be conducted at University of Florida Health Family Medicine clinics. The trial will enroll participants who currently smoke at least 10 cigarettes daily to be randomized into the placebo and AB-free kava arms, being exposed for 4 weeks, with a total of six visits (weeks 0, 1, 2, 4, 8, and 12) to evaluate the compliance and potential issues of AB-free kava use among the participants and to explore the potential effect of the AB-free kava intervention on tobacco dependence, tobacco use, and lung carcinogenesis biomarkers.

### Study participants

Our targeted accrual goal is 80 participants who currently smoke with no intention to quit. Based on previous experience, the rather stringent inclusion/exclusion criteria, and the intensive participation required, the enrollment period is expected to be 3–3.5 years with each participant involved for 13–14 weeks. The sample size was estimated based on urinary TNE data from our pilot trial (urinary TNE is one key endpoint, indicative of the amount of tobacco use) [7]. The mean reduction of TNE after kava treatment is 8245 ng/mg creatinine with a standard error of 9381 ng/mg creatinine, after adjusting for age, sex, race, and smoking status. By assuming the placebo group exhibits at most 15% of the treatment effect, which is a consensus estimate in the literature [14, 15], and the same standard error as in the AB-free kava treatment group, the standardized effect size (Cohen’s *d*) is *d* = 0.75. At alpha of 5%, and using a two-sided Student *t* test, we will achieve 84% power for the primary endpoint (reduction in tobacco use) if the data from 64 participants (32 per group) can be used in the final data analysis. Assuming a 20% attrition rate, which is the rates in similar studies [16], we plan to recruit 80 participants (40 per group).

### Inclusion and exclusion criteria

Eligible participants (a) are adults aged 21 years or above (legal age for smoking in the USA); (b) have self-reported smoking of at least 10 cigarette/day for the past year with no intention to quit; (c) have an expired carbon monoxide level of more than 8 ppm at recruitment; (d) are willing to participate in the study; (e) have access to a functional telephone; (f) are expected to be present in the study’s geographical area for the next 4 months; (g) are not currently enrolled in any smoking cessation programs; and (h) for female subjects of childbearing potential will be required to practice acceptable methods of birth control (the acceptable methods of birth control include birth control pills, birth control shot, birth control implant, IUD, diaphragm, and cervical cap).

Given the proposed mechanism of AB-free kava to facilitate the reduction of tobacco use through the modulation of stress, pregnant women will be excluded from this study because pregnancy is expected to introduce significant changes on mental status and hormone physiology. Such variations are expected to compromise the sample size justification and therefore the primary goal of this early-phase clinical study. There are no additional requirements with respect to gender, employment, geographical, language, or other factors.

The key exclusion criteria are any of the following: (a) diagnosed with cancer (other than non-melanoma skin cancer); (b) diagnosed with liver dysfunction or with previous liver diseases; (c) levels of ALT, AST, ALP, or total bilirubin over limit of normal (ULN) range at prescreen; (d) inability to refrain from acetaminophen, alcohol (no more than one drink daily via self-report), or other potentially hepatotoxic substances; (e) use any other non-cigarette nicotine containing products such as smokeless tobacco, cigar or e-cigarettes; or (f) are pregnant or nursing (lactating) or of childbearing age planning to become pregnant or unwilling to use adequate contraception during the study; or (g) participant answered “Yes” to any of the ASQ questions 1 through 4, or refuses to answer.

### Enrollment and study procedures

Clinic staff will identify potential participants and briefly explain the study. Once a potential participant is identified, clinic staff will refer the potential participant to the study coordinator. The study coordinator will conduct a pre-screening interview to determine the eligibility. Those meeting primary inclusion/exclusion criteria and interested in participation will be scheduled for an in-person assessment. At the assessment visit, participants will be informed and will verbalize understanding that consent is voluntary and that they have the right to withdraw from the study at any time they wish without any consequences to any health care they are receiving currently or in the future. After having their questions answered, if they would like to participate in the study, they will agree to refrain from engaging in any other smoking cessation while participating in this study and sign an informed consent form (Additional file 2: Appendix 1).

Once a participant signs the consent form electronically in REDCap, the participant will complete a breath CO test to evaluate smoking status. Upon confirming tobacco use, normal liver function, no alcohol abuse (via self-report and questionnaires), qualified participants will set up visit 1 (typically within one week) and be randomized into one of the two study arms. During each of the following visits, participant will continue to be monitored with liver function blood tests, a breath CO test, and questionnaires. An additional blood sample (10 mL) and 24-h urine (collected the day before the visit, patients will be provided collection container during each visit) will be used for biomarker assays. The participant then will be provided with medications, written instruction of use, and supplies for the next 24-h urine collection and scheduled for the next visit. Participants will be asked to keep track of missed dose and to return the medication vial at each visit.

### Randomization

Participants who meet eligibility criteria will be randomized to the AB-free kava or the placebo group using a 1:1 allocation scheme, stratified by age, gender, and heavy/light consumption of cigarettes per day. Heavy consumption refers to smoking 25 or more cigarettes per day while light consumption refers to the use of less than 25 cigarettes a day [17]. To be specific, for every 4 participants within each stratification, we will randomly assign 2 of them into the AB-free kava group and the other 2 into the placebo group. Such a stratified block randomization (block size = 4) will ensure a balanced sample size across groups within each stratification over time. This randomization scheme will be implemented by the lead biostatistician in REDCap; after the coordinator enters the age/gender/heavy and light cigarette consumption information, the REDCap will generate a unique identifier. The biostatistician will have provided the underlying treatment group behind these identifiers to the independent Investigational Drug Service (IDS) at UF before the study starts via a pre-established contract. The participant will get the treatment with the identifier from the IDS pharmacist.

Upon completion of study enrollment, IDS will provide the randomization record to the investigative team to unblind assignment. As IDS will operate independently of the study team, the study will be a double-blind trial.

Unblinding can only be performed by IDS and in cases of medical emergencies. The study team does not have to know what arm the participant was assigned to. The reason for unblinding and name of the person who unblinded the participant will be recorded.

### Interventions

AB-free kava and placebo will be obtained from Thorne Research Inc. Chemistry, Manufacturing and Controls (CMC) data have been reviewed by Food and Drug Administration (FDA) with an enabled Investigational New Drug (IND #157256). Such AB-free kava capsules have been analyzed in-house as well for its abundance of kavalactones and lack of flavokavains A and B by our established LC-MS/MS method [18]. AB-free kava or placebo will be administered as follows: one capsule by mouth ideally with meals three times a day (around 8:00 a.m., 1:00 p.m., and 6:00 p.m.) for 28 days. This will result in a daily dose of 225 mg kavalactones for AB-free kava group participants. If a dose is missed, participant will not double the dose at the next scheduled time but document the missed dose and return the missed dose at the next visit.

### Outcomes

The primary objective of this study is to determine whether individuals randomized to the AB-free kava group experience a decrease in the urge to smoke, thereby making it an effective smoking cessation treatment. The primary outcomes will be determined by detecting tobacco use using urinary TNE and questionnaires that ask about participants’ smoking urges and habits. The change in participants’ results and answers compared to baseline will be assessed. A CO test will also be performed at every visit. Urinary TNE and CO levels are useful tools to monitor tobacco use. Urinary TNE level is equivalent to around 90% of a nicotine dose [19] and CO measurement has long been used as a tool to predict smoking habits.

The secondary outcomes include possible reduced lung carcinogenesis that is determined by assessing lung carcinogenesis risk biomarkers (urinary 3-mA and urinary NNAL) and mechanism-based biomarkers (plasma PRKACA, plasma cortisol, and urinary TCE/total cortisol equivalents). Lung cancer chemoprevention was identified in our pilot clinical trial that studied kava’s effect on NNK metabolism. The results showed an increase in urinary excretion of NNAL, a major metabolite of NNK that can cause DNA damage when bioactivated [11]. Cortisol levels are also being measured because our pilot study showed that stress reduction can be a potential mediator for the effect of AB-free kava on tobacco use reduction. Another secondary outcome is providing knowledge about participant compliance and the feasibility of AB-free kava use. This is evaluated in three ways as was done in the pilot trial [7]: (i) self-report of missed doses from participant diary; (ii) returned pill counts by IDS pharmacy; and (iii) urinary detection of dihydromethysticin (DHM) in participants during AB-free kava exposure since DHM is kava-specific. All outcomes will be assessed at each visit.

In addition, this study is expected to make a significant contribution to the literature in the field of supportive care regardless of the trial findings. Indeed, it will fill a vital gap in the literature regarding the ability of kava to reduce the urge to smoke as well as carcinogens in the body. Finally, this study will yield information that will serve as the basis for conducting a follow-up, phase III randomized control trial.

### Ancillary and post-trial care

Major adverse events are not expected from this trial. As such, there is no compensation to participants who may be harmed from trial participation.

### Possible discomforts and risks

Possible discomforts and risks from taking kava supplement are minimal. Preliminary data from a previous human study did not report any significant adverse events due to the consumption of kava supplementation [18]. Because there is a potential for kava to affect the function of the liver through drug-herb interaction (< 0.3 cases per million dosages) [20–28], we will exclude any subjects with liver conditions and will monitor liver function during and after participants are receiving the intervention. AB-free kava (a kava preparation free of flavokavains A and B, the potential hepatotoxic ingredients in kava [29]) will be used in the study as well, which is expected to have an improved safety profile. To minimize and closely monitor any potential adverse events/unanticipated problems, we will exclude individuals with elevated risk for hepatotoxicity, provide additional education, and closely monitor liver functions during the treatment period with 4 and 8-week follow-ups, which will be sufficient even based on kava’s reported hepatotoxic cases [30]. Specifically, participants will be asked to control alcohol use (1 drink per day) and refrain from products containing acetaminophen through the study period. They will be provided with a reference list of medications that contain acetaminophen. ALT, AST, ALP, and total bilirubin will be assessed at every visit to ensure any adverse effects/risks identified and addressed.

Alcohol use will be assessed via questionnaires (self-report). A hepatologist with clinical expertise in liver safety monitoring will supervise liver screening and safety monitoring. In the event that serum liver enzymes become elevated (3 times the normal range, which is considered as mild elevation clinically), participants will be notified immediately and retested in the next 48–72 h. If increases to > 5 x ULN, the study drug will be discontinued and participant referred for further clinical evaluation and treatment. For any increase in ALT, AST, ALP, or total bilirubin > 3 x ULN associated with fatigue, nausea, vomiting, right upper quadrant pain or tenderness, fever, and/or rash, the study drug will be discontinued and participant referred for further clinical evaluation and treatment. The hepatologist will report any adverse events to DISC.

There is minimal risk associated with blood draws, which will be performed by certified professionals.

Similarly, there are potential risks to confidentiality for participants in this study. However, these risks are minimal given our procedures to protect against these risks. Any concerns related to the intervention and privacy will be shared with the PI and every effort will be made to protect the participants’ information during the study. All study personnel are accustomed to maintaining and ensuring patient confidentiality in the course of their work. In addition, all study personnel are highly trained in research procedures and in issues regarding protection of participants’ rights and privacy. All members of the research team, including the PI, coinvestigators, study coordinator, data collectors, research assistants, data manager, and all students associated with the project will complete mandated human subject training prior to study commencement.

Study participants are expected to encounter no other discomforts and risks (physical, psychological, social, and/or economic).

Given kava’s potential neurological functions, we have built in an Ask Suicide-Screening Questions (ASQ) form to assess the suicide risk. This questionnaire will be evaluated in real time by the study physician and can refer the patient to mental health services if needed. However, the probability of suicide risk is extremely low based on our current knowledge of kava use [11]. Should such an emergency arise (statement or demonstration of active suicidal ideation), standard of care clinic procedures will be followed. Specifically, Alachua County Crisis Center would be called to have the patient evaluated in clinic or the patient would be sent directly to a psychiatric facility for further evaluation and management either voluntarily or under a Baker Act. Additionally, all participants will be provided with resources for mental health help. The following resources will be provided to patients in the ICF as well as upon request, UF psychiatry clinical sites, the Alachua County Crisis Center, and the National Suicide Hotline.

In addition, kava use may result in sedation that the participant should not operate heavy instruments and should drive with care within 2 h after the first AB-free kava use, until they know how it affects them although such a risk is extremely low at the proposed dosage regimen. Additionally, our clinical team has ample experience to protect against or minimize potential unexpected discomforts and risks. There are also no potential financial risks that study participants may incur given the trial is free of charge to participants with all supplies provided and incentives are provided as well.

### Participant timeline

**Fig. 1:**
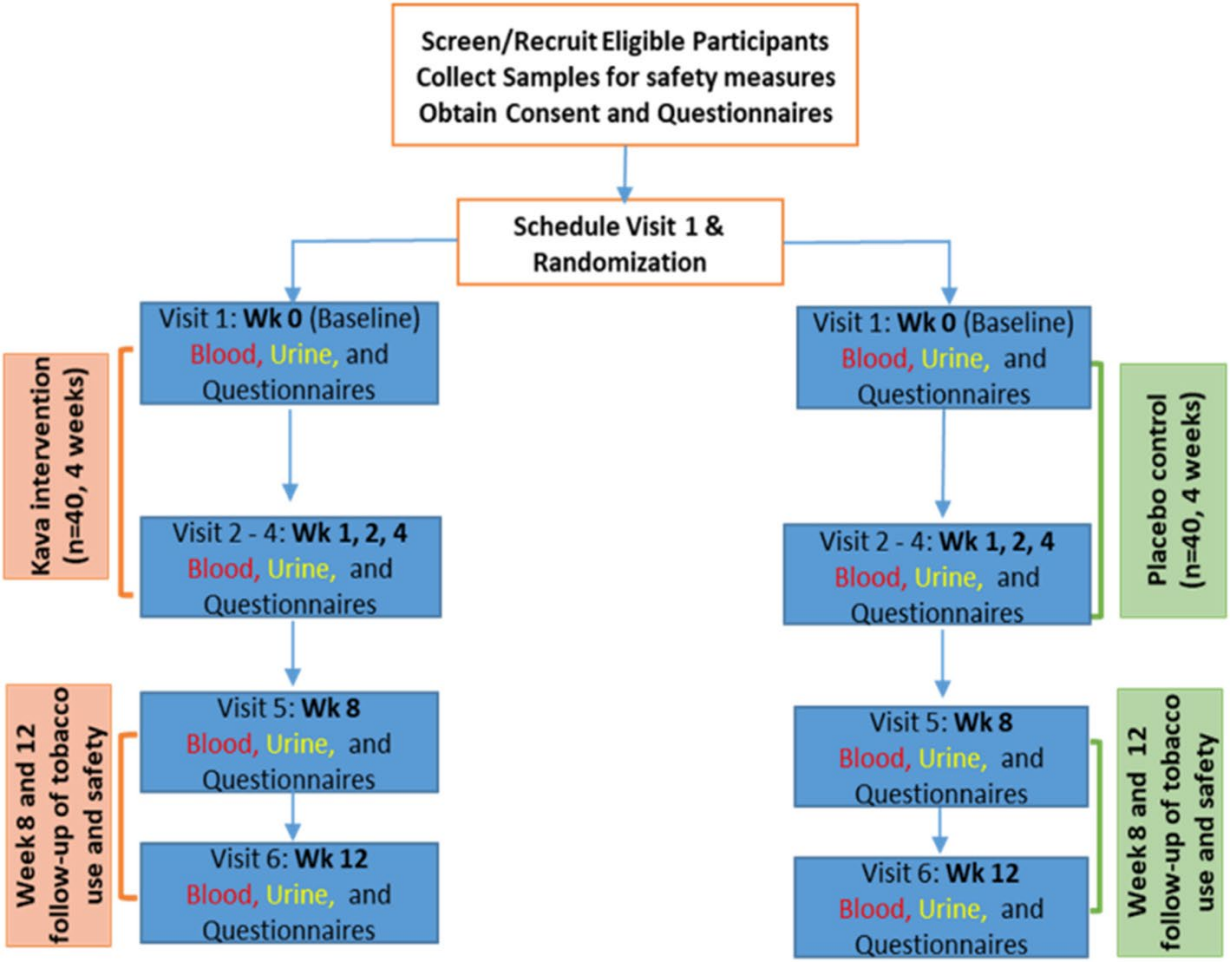
Study design of 4-week AB-free intervention, two follow-ups, and data collection

This is a double-blind randomized placebo-controlled intervention trial. Methods of the protocols have been established in the study laboratories to adequately answer the questions addressed in the objectives. Placebo or AB-free kava intervention are 4 weeks long with one capsule each time and three times daily administered orally. The dose of AB-free kava will be 75 mg of kavalactones per capsule. There will be no dose modification and the use will discontinue if severe adverse effect is detected, which is detailed later (Fig. 1).

### Experimental procedures

During each of following visits 1–6 (each should take about 30–60 min), participants will be screened for ALT, AST, ALP, total bilirubin, breath CO test, and questionnaires administered. An additional blood sample (10 mL, onsite) and 24-h urine (participants will be provided the collection container during each visit and will bring their urine samples to their next visit) will be used for biomarker assays. The participant then will be provided with medications and written instruction of use, supplies for the next 24-h urine collection, and scheduled for the next visit.

At the end of the study or when a participant drops out of the study, an exit interview will be performed with open-ended questions to obtain feedbacks about the trial from the participants.

### Data and biological sample collection and storage

Participants’ identities will be protected through the following measures: consent forms will be stored behind a locked door and in a locked filing cabinet accessible only by the study team, and all identifying information will be stored in a separate, secured location from data that is collected during the study (Additional file 2: Appendix 2). Access to locked files will be restricted to essential personnel only. In addition to consent and identification files that are secured, all data files will be coded in a manner that makes identification of the participants extremely unlikely. Indeed, participants will be given a numerical ID code to ensure that inadvertent or unauthorized identification does not occur. Data will receive additional layers of security because the whole building, floor, and office in which locked records are maintained have restricted, keyed access. The anonymous codes assigned to participants will be verified and maintained throughout the study, and codes with names will be provided only to meet federal guidelines applicable to the facility.

All data (participant information, questionnaire responses, values of biological samples and others) will be stored in a REDCap (Research Electronic Data Capture) database.

Initial examination of data will include descriptive statistics, frequency distributions, and histograms to identify outliers, missing data, and to check data source adequacy. Checks for implausible or out-of-range values, distributional forms, and missingness will be performed. Any entry error and/or inconsistency will be discussed during meetings with the study team.

Quarterly statistical summaries and progress reports will be reviewed by the study investigators. To ensure participant confidentiality, all personal identifiers will be expunged from the data set.

Our research coordinator will conduct the screening of potential participants working closely with the clinical staff. The investigative team will meet weekly to discuss study progress.

An environmental assessment is not required because the action requested qualifies for a categorical exclusion per 21 CFR 25.31(e). To the applicant’s knowledge, no extraordinary circumstances exist per 21 CFR 25.15(d).

For biological samples, a research assistant will retrieve the urine and blood samples from the clinic within 1 h after the participant’s visit and deliver to the study’s laboratory. The total volume of the urine sample will be recorded to the closest 10 mL. The urine samples will be split into 5 × 15 mL and stored at – 80 °C. The blood samples will be processed into plasma, buffy coat, and red blood cells and stored at – 80 °C. Detailed information of these samples, including participant ID, visit #, sample information, and missed dosing or sample(s), will be recorded in the REDCap database.

Supplies for each participant will be prepared by IDS following randomization. Instructions on use and storage will be provided in writing. Unused materials will be returned at the next visit, recorded, and destroyed by IDS. IDS will maintain a Study Drug Accountability log for each participant, which will record participant ID, randomization code, medication ID, dates of medication dispensed, returned, missed dose, number of capsules returned, and medication destruction.

### Participant retention

The study team has an excellent history of retaining clinical trial participants. We will always use respectful, empathetic communication and be considerate of participants’ time when scheduling research activities. Compliance and retention will be maximized via the following methods: (a) close monitoring during participation; (b) a reminder phone call to confirm study visits; (c) 24/7 access to the study team during participation; and (d) establishing appropriate rapport such that the participant feels motivated to return. Participant will also receive a gift card for each visit as compensation. These strategies should minimize missing data and improve data validity.

### Questionnaires

During each visit, the participants will be administered questionnaires (Additional file 1) that include the following: (1) Self-Reported Measure of Smoking, documenting the number of cigarettes smoked in the past 7 days. (2) Fagerström Test for Nicotine Dependence (FTND) [31], a 6-item scale measuring the level of nicotine dependency or addiction. It assesses how soon tobacco use begins each day, which cigarettes during the day a person could do without, how adults who smoke cope in places where they cannot smoke, and how frequently and how deeply they smoke. (3) Modified Cigarette Evaluation Questionnaire (mCEQ) [32]: a 12-item scale, rating several dimensions (satisfaction, psychological reward, nausea or dizziness, craving relief; enjoyment of airway sensations). It assesses the degree to which participants experience the reinforcing effect of smoking. (4) Brief Questionnaire on Smoking Urges (QSU-Brief) [33]: a 10-item scale, assessing the features of craving, including the anticipation of relief of nicotine withdrawal, anticipation of positive outcomes of smoking, desire to smoke, and intention to smoke. (5) Perceived Stress Scale (PSS) [34]: a 10-item scale measuring the perception of stress. It is a measure of the degree to which situations in one’s life are appraised as stressful. The scale also includes a number of direct queries about current levels of experienced stress. (6) Insomnia Severity Scale [35]: a 7-item self-report questionnaire assessing the nature, severity, and impact of insomnia. Dimensions evaluated are as follows: severity of sleep onset, sleep maintenance, and early morning awakening problems, sleep dissatisfaction, interference of sleep difficulties with daytime functioning, noticeability of sleep problems by others, and distress caused by the sleep difficulties. Because of kava’s potential benefit to sleep, this questionnaire will generate preliminary data about kava’s mechanism in facilitating tobacco cessation. (7) Ask Suicide-Screening Questions (ASQ) [36]: consists of four yes/no questions to identify individuals that require further mental health/suicide safety assessment. According to FDA recommendation for any interventions with neurological activities, ASQ is included to assess suicide risk because of its peer-reviewed effectiveness and the lower burden on the participants.

### Statistical analysis

#### Principle

All data (questionnaires and biomarkers) will be checked for out-of-range values and normality. Decisions regarding parametric vs nonparametric statistics and data transformation will be based on such results. Participants who received at least one dose of AB-free kava treatment or placebo and have at least one more visit beyond the baseline visit will be considered as evaluable.

#### Missing data consideration

We expect to have three scenarios for participants with respect to missing data: (1) participants complete all visits and have no missing outcomes. They will be included in the statistical analysis. (2) Some participants may not complete all visits but have outcomes for at least one visit after the baseline visit. These participants will be included in the statistical analysis as well. The missing outcomes will be handled by either the Last Observation Carry Forward (LOCF) technique or linear mixed model for longitudinal data analysis (see below for details). (3) Participants do not complete the first two visits after randomization. They will be excluded from data analysis and we will replace these participants (also see the “Replacement plan” section). An enrolled participant will be replaced if s/he (i) is not involved in the baseline visit or (ii) is only involved in the baseline visit and not in any of the next five visits. Such cases are expected to be no more than 20%.

#### Account for spurious data

All data (questionnaires and biomarkers) will be checked for out-of-range values and normality. If any spurious data (outliers) were identified, we will fit nonparametric statistical models to assess the robustness of our primary parametric data analysis.

#### Replacement plan

If a participant for any reason leaves the study without completing the first two visits after randomization, the study team will recruit a new participant to replace the study subject that is no longer in the study.

#### Longitudinal data analysis

To study the relationship between longitudinal outcomes and treatment effect, we will fit linear mixed effects models with subject specific random effects to account for within subject correlation, using R software lmer package. Linear mixed model can accommodate missing outcomes if they are missing at random. Baseline outcomes and covariates including age, sex, race, and smoking status will be adjusted to account for confounding effects. Past COVID-19 status may also be a covariate if there are enough cases. Urinary TNE, one key endpoint, will be used to describe how the statistical models test our working hypotheses. The other measurement will follow the same model.

##### Hypothesis 1: active AB-free kava treatment (week 0–week 4) is effective in reducing TNE as compared to the placebo group

To test this hypothesis, we will fit the following linear mixed model:

1 $$y_{it}=\mu+\alpha_i+\lbrack\eta+\beta\mathbb{I}(z_i=1)\rbrack t+\sum\limits_{l=0}^L\gamma_lx_{il}+\varepsilon_{it},\alpha_i\sim N(0,\sigma_0^2),\varepsilon_{it}\sim\;N(0,\sigma^2)$$

where *y*_*it*_ is the outcome variable (e.g., TNE) for subject *i* and week *t* (0 ≤ *t* ≤ 4 weeks); *μ* is the intercept term; ${\alpha}_i\sim N\left(0,{\sigma}_0^2\right)$ is the random effect with variance term ${\sigma}_0^2$ for subject *i*; *z*_*i*_ is the treatment indicator with *z*_*i*_ = 0 for the placebo group and *z*_*i*_ = 1 for the AB-free kava group; *η* is the slope of time effect of the placebo group; *β* is the additional slope of the treatment effect (AB-free kava group compared to placebo group), and $\mathbb{I}$ is the indicator function which equals to 1 if the argument inside (.) is true and equals 0 otherwise; *x*_*il*_ is the *l*^*th*^ covariates for subject *i* with *l* = 0 for baseline level of the outcome measurements (e.g., baseline TNE) and *l* = 1, 2, …, *L* for *L* covariates (e.g., age, sex, race, smoking history, etc.); *ε*_*ik*_~*N*(0, *σ*^2^) is the error term with variance *σ*^2^. In Equation (1), *β* is the additional slope of active AB-free kava effect during week 0 to week 4 compared to the placebo group; we will perform the statistical hypothesis testing (H_0_: *β* = 0) to provide its *p*-value, point estimate and confidence interval. A significant *p*-value will indicate that the AB-free kava treatment significantly reduces TNE during week 0 through week 4 compared to the placebo group. Equation (1) only considers a random intercept, which allows the baseline level of the outcome measurement to be different. We will further incorporate random slope terms in Equation (1), if the slopes of the linear trajectories vary among different individuals, which is likely. In addition, if we observe that the trajectory of AB-free kava’s effect through weeks 0, 1, 2, and 4 is not linear, we will consider to use the observations at week 0 and week 4 to test *Hypothesis 1*. The following linear model will be fitted:

2 $${\Delta y}_i=\mu+\mathbb{I}\left(z_i=1\right)\times\theta+\sum\limits_{l=0}^L\gamma_lx_{il}+\varepsilon_i,\varepsilon_i\sim N\left(0,\sigma^2\right)$$

where Δ*y*_*i*_ is the 4-week TNE change from week 0 for subject *i*; *μ* is the intercept; *θ* is the cumulative active AB-free kava effect in 4 weeks compared to the placebo group. We will perform the statistical hypothesis testing and provide point and confidence interval estimates of the efficacy treatment effect *θ*. If the outcome one visit is missing, we will adopt the last observation carry forward (LOCF) technique to impute [37].

##### Hypothesis 2: AB-free kava has a sustainable effect in reducing TNE in week 4–week 12

The knowledge about AB-free kava’s sustainable effect is critical for its future development and practice. We will test whether the AB-free kava effect (week 0–week 4) can be extended to the follow-up period week 4–week 12, when AB-free kava treatment is absent. The following two sub-hypotheses will be examined:

##### Hypothesis 2a AB-free kava’s effect will sustain to the follow-up period

To test this hypothesis, we will only consider the AB-free kava treatment arm with 4 ≤ *t* ≤ 12 weeks. We will fit the following linear mixed model:

3 $$y_{it}=\mu+\alpha_i+\beta_1\mathbb{I}\left(t=8\right)+\beta_2\mathbb{I}\left(t=12\right)+\sum\limits_{l=0}^L\gamma_lx_{il}+\varepsilon_{it},\alpha_i\sim N\left(0,\sigma_0^2\right),\varepsilon_{it}\sim N\left(0,\sigma^2\right)$$

where *μ* is the AB-free kava treatment effect at week 4; *β*_1_ is the AB-free kava treatment effect at week 8 compared to week 4; *β*_2_ is the AB-free kava treatment effect at week 12 compared to week 4; the rest of the parameters have the same interpretation as Equation (1). In Equation (3), we will perform the statistical hypothesis testing (H_0_: *β*_1_ ≤ 0 vs H_A_: *β*_1_ > 0, and H_0_: *β*_2_ ≤ 0 vs H_A_: *β*_2_ > 0). Rejecting the null hypothesis will imply *β*_1_ > 0 or *β*_2_ > 0, which indicates that the AB-free kava effect is not sustainable. In other words, TNE level will go up and the participants likely relapse. Failing to reject the null hypothesis will imply that the TNE level stays steady and the AB-free kava treatment effect likely sustains through week 8 and week 12. We will provide their *p*-value, point estimates and confidence intervals.

##### Hypothesis 2b the extended AB-free kava effect is superior to placebo

To evaluate this hypothesis, we will directly apply Equation (1) in Hypothesis 1, but treat week 4 as baseline, and week 12 as follow-up. Under this scenario, *β* is the slope of the extended AB-free kava effect (weeks 4–12) compared to the placebo group. We will perform the statistical hypothesis testing (H_0_: *β* = 0) to provide its *p*-value, point estimate and confidence interval. A significant *p*-value will indicate that the extended AB-free kava effect significantly reduces TNE during the follow-up period (week 4 through week 12) compared to the placebo group, indicating AB-free kava’s sustainable effect superior to placebo.

##### Exploratory hypothesis 3: tobacco reduction by AB-free kava is potentially mediated via reductions in plasma PRKACA, cortisol, or urinary TCE

Our pilot data suggested that adults who smoke with higher baseline levels of cortisol appeared to have more reductions in TNE upon AB-free kava use while the extents of reduction in TNE correlate positively with the reductions in plasma cortisol. Although the difference and correlation were not statistically significant, these results support the potential association between stress reduction and tobacco use reduction. We therefore hypothesize that changes in plasma PRKACA, plasma cortisol or urinary TCE are potential mediators for the effect of AB-free kava in reducing TNE. To test this hypothesis, we will perform mediation analysis, where TNE reduction is the outcome variable, AB-free kava intervention is the predictor, plasma PRKACA, cortisol or urinary TCE is the mediator, adjusting for age, sex, race, and smoking status as covariates. The direct effects, indirect effects, and mediation proportion will be determined using the R *mediation* package. Their *p*-values and 95% confidence intervals will be reported. Such hypothesis, if tested positive, will provide mechanistic insight—whether AB-free kava may induce TNE reduction via relieving stress.

##### Exploratory hypothesis 4: baseline levels of plasma cortisol, PRKACA, or urinary TCE can benchmark the efficacy of AB-free kava treatment (precision intervention potential)

To test this hypothesis, we will fit linear mixed models within the AB-free kava group, where the repeated measurement of TNE are the outcome variables, baseline level of plasma cortisol, PRKACA or urinary TCE is the predictor, assuming subject specific random effect, and adjusting for the covariates mentioned previously. If these baseline biomarkers are significant predictors of TNE reduction, we will further dichotomize participants into high/low groups using median (or tertile/quartile) and examine AB-free kava efficacy based on groups determined by these biomarkers. If this hypothesis is tested positive, AB-free kava has the potential of precision intervention with these biomarkers to identify those who will benefit the most of AB-free kava use.

##### Plan for conducting an interim analysis and criteria for stopping rules

When 25% participants complete the study, safety data of all participants will be analyzed by the Data Integrity and Safety Committee independently to determine whether AB-free kava intervention has any potential risk. The same practice will be implemented at 50% and 75% completion. If the adverse events are significantly higher in the AB-free kava arm than the placebo arm, the trial will be suspended to discuss with FDA and Florida Department of Health to determine whether the trial should stop.

### Data and safety monitoring plan

The data and safety monitoring committee consists of physicians, statisticians, pharmacists, and toxicology experts. The research team is responsible for recruiting and following-up with participants, providing them with the trial material, and monitoring and reporting adverse events to the IRB. The research team will be granted access to the final trial dataset.

Once a participant is enrolled, continuous monitoring will be conducted by the investigative team as well as IRB. Any serious adverse events as well as unanticipated problems involving risks to participants or others, will be reported to IRB, FDA, and study sponsor. Our monitoring plan includes the following measures:

1. Monitor adverse events, study progression, and data quality issues; consider factors external to the study when interpreting data, such as new scientific or therapeutic developments that may have an effect on the safety of the participants or the ethics of the study; and maintain confidentiality during all phases of the trial.
2. Participants will be assessed for ALT, AST, ALP, and total bilirubin at baseline and weeks 1, 2, 4, 8, and 12 to monitor potential acute or delayed hepatotoxic risk. Participants, whose ALT, AST, ALP, or total bilirubin increases > 3 x the upper limit of normal (ULN) but ≤ 5 x ULN, will retest after 48 to 72 h. If increases to > 5 x ULN, the study drug will be discontinued and participant referred for further clinical evaluation and treatment. For any increase in ALT, AST, ALP, or total bilirubin > 3 x ULN associated with fatigue, nausea, vomiting, right upper quadrant pain or tenderness, fever, and/or rash, the study drug will be discontinued and participant referred for further clinical evaluation.
3. The study will undergo regular audits by a Data Integrity and Safety Committee (details below).

### Data integrity and oversight

Per UF IRB requirements, the PI will be personally responsible for conducting and supervising the conduct of human subject research by “protecting the rights, safety, and welfare of subjects under the investigator’s care.” The PI will also ensure that all the research conducted is done so in an ethical manner and in accordance with all federal, state, and local laws and regulations, institutional policies, and the requirements of the IRB. The PI has voluntarily accepted these responsibilities and will fully comply with these requirements, as outlined in the UFHCC Guidance: Principal Investigator Responsibilities and Oversight.

The PI will be primarily responsible for continuous monitoring of adverse events, unanticipated problems, protocol violations, and other immediate protocol issues. The PI or their designee will collect information on subjects enrolled through the use of electronic or paper source documents, CRFs, and informed consent forms.

The PI will be responsible for ensuring the accuracy, completeness, legibility, and timeliness of the data reported. All source documents will be completed in a neat, legible manner to ensure accurate interpretation of data. The study team will maintain adequate case histories of study subjects, including accurate case report forms (CRFs), and source documentation. In case protocol modification is required, a notice will be sent to the IRB, sponsor, as well as the researchers and participants.

### Data integrity and safety committee (DISC)

This protocol will be reviewed and monitored by the University of Florida Health Cancer Center Data Integrity and Safety Committee (UFHCC DISC) in accordance with their policies and procedures. They will review and monitor study progress, toxicity, safety, and other data from this trial. Questions about subject safety or protocol performance will be addressed with the sponsor-investigator, statistician, and study team members. Should any major concerns arise, the DISC will offer recommendations regarding whether or not to suspend the trial.

UFHCC DISC data and safety monitoring activities include:

- Review of clinical trial conducted for progress and safety
- Review of all adverse events requiring expedited reporting as defined in the protocol
- Review of reports generated by data quality control review process
- Notification of the sponsor-investigator of recommended action
- Notification of sites coordinated by the UFHCC of adverse events requiring expedited reporting and subsequent committee recommendations for study modifications

In compliance with the UFHCC data and safety monitoring plan, the PI will provide a Data Integrity and Safety Committee Report to DISC at the predetermined timelines for the level of risk category assigned during the initial SRMC CCPSP (Scientific Review and Monitoring Committee Cancer Control and Population Sciences Panel) review, which occurs prior to initial IRB approval.

UFHCC investigator-initiated protocols will be classified into one of the following categories of risk by the SRMC CCPSP (see *SRMC manual* for further details):

Level 1 – *Low risk* Investigator Initiated interventional trials

Level 2 – *Moderate risk* Investigator Initiated or externally sponsored interventional (such as drug, biologic or device) trials using FDA approved or commercially available compounds or interventions

Level 3 – *High risk* Investigator Initiated or externally sponsored interventional trials (such as investigator-sponsored INDs, phase I trials, studies requiring biosafety approval, or other areas that may be designated by NIH as high risk)

Level 4 – Complex trials involving *very high risk* to subjects and a high level of complexity such as first in human or gene transfer studies

The risk level assigned by the SRMC CCPSP will determine the appropriate level of DISC monitoring required, with increased monitoring required for higher-risk trials.

### Adverse events/unanticipated problems

To minimize and closely monitor any potential adverse events/unanticipated problems, we will exclude individuals with elevated risk for hepatotoxicity, provide additional education, and closely monitor liver functions during the treatment period with 4- and 8-week follow-ups, which will be sufficient even based on kava’s reported hepatotoxic cases [30]. Specifically, participants will be asked to control alcohol use and refrain from products containing acetaminophen through the study period. They will be provided with a reference list of medications that contain acetaminophen. ALT, AST, ALP, and total bilirubin will be assessed at every visit to ensure any adverse effects/risks identified and addressed. Alcohol use will be assed via questionnaires (self-report). A hepatologist with clinical expertise in liver safety monitoring will supervise liver screening and safety monitoring. Participants with an increase in liver biochemistry will be referred to clinical services for further evaluation to minimize the risk with plan detailed below. The hepatologist will report any adverse events to DISC.

Any concerns related to the intervention and privacy will be shared with the PI and every effort will be made to protect the participants’ information during the study. All study personnel are accustomed to maintaining and ensuring patient confidentiality in the course of their work. In addition, all study personnel are highly trained in research procedures and in issues regarding protection of participants’ rights and privacy. All members of the research team, including the PI, coinvestigators, study coordinator, data collectors, research assistants, data manager, and all students associated with the project will complete mandated human subject training prior to study commencement.

### Dissemination plan

All results will be reported in ClinicalTrials.gov. The full protocol, statistical code, and participant dataset are available from the corresponding author upon request.

Trial results will be published in peer-reviewed scientific journals with open-access to guarantee wide distribution. Findings will also be communicated to the public via presentations at conferences.

## Trial status

Protocol version number 0001.3. Date recruitment began: 03/02/2022. Approximate date when recruitment will be completed: 11/2025. https://trialsearch.who.int/Trial2.aspx?TrialID=NCT05081882

## Schedule of events

**Table:** 

Visit: Procedure:	Screening (≤ 21 days prior to day 1)	Baseline (day 1)	Week 1 (day 8 ± 3 days)	Week 2 (day 15 ± 3 days)	Week 4 (day 29 ± 3 days)	Week 8 follow-up (day 57 ± 3 days)	Week 12 follow-up (day 85 ± 3 days)
Informed consent	**X**						
Demographic information	**X**						
Medical history	**X**						
Brief physical exam	**X**				**X**		
CO breath test	**X**	**X**	**X**	**X**	**X**	**X**	**X**
Height	**X**						
Weight	**X**						
Biomarker assessment-(blood and urine)		**X**	**X**	**X**	**X**	**X**	**X**
Labs (CMP, CBC w diff)	**X**	**X**	**X**	**X**	**X**	**X**	**X**
Pregnancy test (urine)	**X**						
Randomization	**X**						
Administration of study drug		**X**	**X**	**X**			
Review and/or dispense subject diary		**X**	**X**	**X**	**X**		
Concomitant medication review	**X**	**X**	**X**	**X**	**X**		
Adverse event review		**X**	**X**	**X**	**X**	**X**	**X**
24-h urine supplies	**X**	**X**	**X**	**X**	**X**	**X**	
Questionnaires	**X** ^**a**^	**X**	**X**	**X**	**X**	**X**	**X**

***Insert information, as appropriate, if superscripts are used within the table. Examples provided below.***

*Abbreviations*: *H&P* History and Physical examination, *PS* ECOG performance status, *VS* vital signs (blood pressure, temperature, pulse and respiratory rates, weight and height), *TOX* toxicity assessment, *CBC/diff* complete blood count and white blood cell differential, *CMP* 12 item complete metabolic profile (sodium, potassium, chloride, bicarbonate, blood urea nitrogen, creatinine, glucose, alkaline phosphatase, AST, ALT, total bilirubin), *UA* urinalysis

^a^ASQ questionnaire only, given at screening

## Supplementary Information

**Additional file 1.** Measures for tobacco use, craving, addiction, stress, and insomnia: include the questionnaires. Schedule of events.

**Additional file 2: Appendix 1.** Informed Consent. **Appendix 2.** Biological Specimens.
