# Supplementary material for: Reducing tobacco-associated lung cancer risk: a study protocol for a randomized clinical trial of AB-free kava
Source: Trials. 2023 Jan 18;24:36. doi: 10.1186/s13063-023-07081-x (PMC9847434; doi:10.1186/s13063-023-07081-x)

**1. Measures for tobacco use, craving, addiction, stress, and insomnia**

Self-Reported Measures of Smoking Cigarettes

To help us evaluate your cigarette use, we need to get an idea of what your smoking was like in the past _________ days. To do this, we would like you to fill out the attached calendar. Filling out the calendar is not hard! Try to be as accurate as possible. We recognize you won’t have perfect recall. That’s **OKAY**.

**WHAT TO FILL IN**

The idea is to record how many cigarettes you smoked for each day on the calendar. On days when you did not smoke cigarettes, not even one, you should write a "0." It’s important that something is written for every day, even if it is a 0".

**YOUR BEST ESTIMATE**

We realize it isn’t easy to recall things with 100% accuracy. If you are not sure whether you smoked 15 or 16 cigarettes or whether you smoked on a Thursday or a Friday, give it your best guess! What is important is that 15 or 16 cigarettes is very different from 1 cigarette. The goal is to get a sense of how frequently you smoked, how much you smoked, and your patterns of smoking.

**HELPFUL HINTS**

- If you have an appointment book, you can use it to help you recall your use.
- Holidays such as Thanksgiving and Christmas are marked on the calendar to help you recall your smoking. Also, think about how much you smoked on personal holidays & events such as birthdays, vacations, or parties.
- If you have regular patterns to your smoking, you can use these to help you recall your use. For example, some people may only smoke during social situations.

**COMPLETING THE CALENDAR**

A blank calendar is attached. Write in the number of cigarettes you smoked on each day. The time period we are talking about on the calendar is from______________ to __________________. In estimating the number of cigarettes you smoked, be as accurate as possible.

**DOUBLE CHECK THAT ALL DAYS ARE FILLED IN BEFORE RETURNING THE CALENDAR.** Before you start look at the **SAMPLE CALENDAR**.

**Fagerstrom Test for Nicotine Dependence**

1. How soon after you wake up do/did you smoke your first cigarette?
   1. Within 5 minutes [3 points]
   2. 6-30 minutes [2 points]
   3. 31-60 minutes [1 point]
   4. After 60 minutes [0 points]
2. Do/Did you find it difficult to refrain from smoking in places where it is forbidden, e.g., in church, at the library, in a cinema, etc.?
   1. Yes [point]
   2. No [0 points]
3. Which cigarette would you hate most to give up?
   1. The first one in the morning [1 point]
   2. All others [0 points]
4. How many cigarettes per day do/did you smoke?
   1. 10 or less [0 points]
   2. 11-20 [1 point]
   3. 21-30 [2 points]
   4. 31 or more [3 points]
5. Do/did you smoke more frequently during the first hours after waking than during the rest of the day?
   1. Yes [1 point]
   2. No [0 points]
6. Do/Did you smoke if you are so ill that you are in bed most of the day?
   1. Yes [1 point]
   2. No [0 points]

**Modified Cigarette Evaluation Questionnaire**

If you have smokes since you last completed this questionnaire, please mark the number that best represents how smoking made you feel (1-not at all, 2-very little, 3-a little, 4-moderately, 5-a lot, 6-quite a lot, 7-extremely).

1. Was smoking satisfying?
2. Did cigarettes taste good?
3. Did you enjoy the sensations in your throat and chest?
4. Did smoking calm you down?
5. Did smoking make you feel more awake?
6. Did smoking make you feel less irritable?
7. Did smoking help you concentrate?
8. Did smoking reduce your hunger for food?
9. Did smoking make you dizzy?
10. Did smoking make you nauseous?
11. Did smoking immediately relieve your craving for a cigarette?
12. Did you enjoy smoking?

**Brief Questionnaire on Smoking Urges**

Indicate how much you agree or disagree with each of the following statements by placing a single checkmark (like this: [✔]) by a number ranging from 1 (strongly disagree) to 7 (strongly agree). The closer you place your checkmark to one end or the other indicates the strength of your disagreement or agreement. Please complete every item. We are interested in how you are thinking or feeling right now as you are filling out this questionnaire.

1. I have a desire for a cigarette right now.

Strongly Disagree 1[ ] 2[ ] 3[ ] 4[ ] 5[ ] 6[ ] 7[ ] 8[ ] 9[ ] 10[ ] Strongly Agree

1. Nothing would be better than smoking a cigarette right now.

Strongly Disagree 1[ ] 2[ ] 3[ ] 4[ ] 5[ ] 6[ ] 7[ ] 8[ ] 9[ ] 10[ ] Strongly Agree

1. If it were possible, I probably would smoke right now.

Strongly Disagree 1[ ] 2[ ] 3[ ] 4[ ] 5[ ] 6[ ] 7[ ] 8[ ] 9[ ] 10[ ] Strongly Agree

1. I could control things better right now if I could smoke.

Strongly Disagree 1[ ] 2[ ] 3[ ] 4[ ] 5[ ] 6[ ] 7[ ] 8[ ] 9[ ] 10[ ] Strongly Agree

1. All I want right now is a cigarette.

Strongly Disagree 1[ ] 2[ ] 3[ ] 4[ ] 5[ ] 6[ ] 7[ ] 8[ ] 9[ ] 10[ ] Strongly Agree

1. I have an urge for a cigarette.

Strongly Disagree 1[ ] 2[ ] 3[ ] 4[ ] 5[ ] 6[ ] 7[ ] 8[ ] 9[ ] 10[ ] Strongly Agree

1. A cigarette would taste good now.

Strongly Disagree 1[ ] 2[ ] 3[ ] 4[ ] 5[ ] 6[ ] 7[ ] 8[ ] 9[ ] 10[ ] Strongly Agree

1. I would do almost anything for a cigarette now.

Strongly Disagree 1[ ] 2[ ] 3[ ] 4[ ] 5[ ] 6[ ] 7[ ] 8[ ] 9[ ] 10[ ] Strongly Agree

1. Smoking would make me less depressed.

Strongly Disagree 1[ ] 2[ ] 3[ ] 4[ ] 5[ ] 6[ ] 7[ ] 8[ ] 9[ ] 10[ ] Strongly Agree

10. I am going to smoke as soon as possible.

Strongly Disagree 1[ ] 2[ ] 3[ ] 4[ ] 5[ ] 6[ ] 7[ ] 8[ ] 9[ ] 10[ ] Strongly Agree

**Perceived Stress Scale**

For each question choose from the following alternatives:

1. Never 1- Almost Never 2- Sometimes 3- Fairly Often 4- Very Often
2. _____ In the last month, how often have you been upset because of something that happened unexpectedly?
3. _____ In the last month, how often have you felt that you were unable to control the important things in your life?
4. _____ In the last month, how often have you felt nervous or stressed?
5. _____ In the last month, how often have you felt confident about your ability to handle your personal problems
6. _____ In the last month, how often have you felt that things were going your way?
7. _____ In the last month, how often have you found that you could not cope with all the things that you had to do?
8. _____ In the last month, how often have you been able to control irritations in your life?
9. _____ In the last month, how often have you felt that you were on top of things?
10. _____ In the last month, how often have you been angered because of things that happened that were outside of your control?
11. _____ In the last month, how often have you felt difficulties were piling up so high that you could not overcome them?

**Insomnia Severity Index**

The Insomnia Severity Index has seven questions. The seven answers are added up to get a total score. When you have your total score, look at the 'Guidelines for Scoring/Interpretation’ below to see where your sleep difficulty fits.

For each question, please circle the number that best describes your answer.

Please rate the current (i.e. last 2 weeks) severity of your insomnia problem(s).

| **Insomnia Problem** | **None** | **Mild** | **Moderate** | **Severe** | **Very Severe** |
| --- | --- | --- | --- | --- | --- |
| 1. Difficulty falling asleep | 0 | 1 | 2 | 3 | 4 |
| 2. Difficulty staying asleep | 0 | 1 | 2 | 3 | 4 |
| 3. Problems waking up too early | 0 | 1 | 2 | 3 | 4 |

4. How SATISFIED/DISSATISFIED are you with your current sleep pattern?

| Very Satisfied | Satisfied | Moderately Satisfied | Dissatisfied | Very Dissatisfied |
| --- | --- | --- | --- | --- |
| 0 | 1 | 2 | 3 | 4 |

1. How NOTICEABLE to others do you think your sleep problem is in terms of impairing the quality of your life? Not at all.

| Noticeable | A Little | Somewhat | Much | Very Much Noticeable |
| --- | --- | --- | --- | --- |
| 0 | 1 | 2 | 3 | 4 |

1. How WORRIED/DISTRESSED are you about your current sleep problem?

| Worried | A Little | Somewhat | Much | Very Much Worried |
| --- | --- | --- | --- | --- |
| 0 | 1 | 2 | 3 | 4 |

1. To what extent do you consider your sleep problem to INTERFERE with your daily functioning (e.g. daytime fatigue, mood, ability to function at work/daily chores, concentration, memory, mood, etc.) CURRENTLY?

| Interfering | A Little | Somewhat | Much | Very Much Interfering |
| --- | --- | --- | --- | --- |
| 0 | 1 | 2 | 3 | 4 |

**Guidelines for Scoring/Interpretation**

Add the scores for all seven items (questions 1 + 2 + 3 + 4 + 5 +6 + 7) =

Total score categories:

0–7 = No clinically significant insomnia

8–14 = Subthreshold insomnia

15-21 = Clinical insomnia (moderate severity)

22-28 = Clinical insomnia (severe)

**Ask Suicide-Screening Questions (ASQ)**

*Used via courtesy of* [*www.myhealth.va.gov*](http://www.myhealth.va.gov/) *with permission from Charles M. Morin, Ph.D., Université Laval*


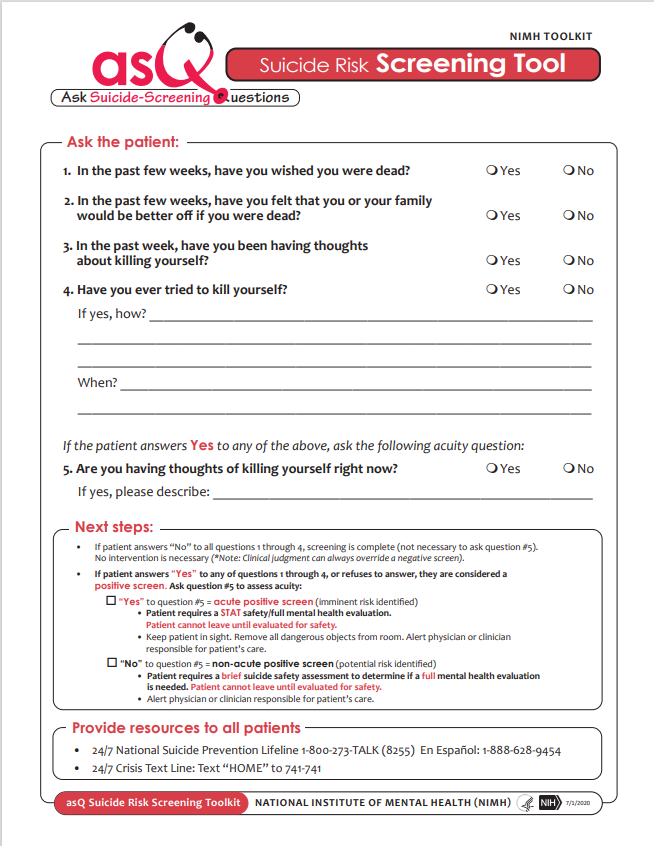

Supplement: Supplementary file 1 — Additional file 1. Measures for tobacco use, craving, addiction, stress, and insomnia: include the questionnaires. Schedule of events. [file 13063_2023_7081_MOESM1_ESM.docx]
