# Supplementary material for: Reducing tobacco-associated lung cancer risk: a study protocol for a randomized clinical trial of AB-free kava
Source: Trials. 2023 Jan 18;24:36. doi: 10.1186/s13063-023-07081-x (PMC9847434; doi:10.1186/s13063-023-07081-x)
Supplement: Supplementary file 2 — Additional file 2: Appendix 1. Informed Consent. Appendix 2. Biological Specimens. [file 13063_2023_7081_MOESM2_ESM.docx]

**Appendix 1: Informed Consent**


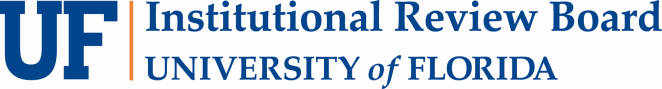


***Informed Consent Form***

***to Participate in Research, and***

***Authorization***

***to Collect, Use, and Disclose Protected Health Information (PHI)***

| **Introduction** |
| --- |

Name of person seeking your consent:

Place of employment & position:

| General Information about this Study |
| --- |

1. Name of Participant ("Study Subject")

___________________________________________________________________

2. What is the Title of this research study (this “Research Study”)?

Reducing tobacco-associated lung cancer risk: A randomized clinical trial of AB-free kava

3. Whom do you call if you have questions about this Research Study (the “Study Team”)?

Principal Investigator: Ramzi Salloum, Ph.D, 352-294-4997

Study Coordinator: Anna Maria Abi Nehme, 352-627-9144, UF-KavaStudy@ufl.edu

4. Who is paying for this Research Study?

The sponsor of this study is Florida Department of Health.

**5. In general, what do you need to know about this Research Study?**

Agreeing to become involved in any research is always voluntary. By signing this form, you are not waiving any of your legal rights. If you decide not to participate in this research, you will not be penalized in any way and you will not lose any benefits to which you are entitled. If you have questions about your rights as a research subject, please call the University of Florida Institutional Review Board (IRB) office at (352) 273-9600.

1. **In general, what is the purpose of the research, how long will you be involved?**

Tobacco use is the leading cause of many preventable diseases, particularly lung cancer. Based on the national cancer data in 2020, Florida has the highest lung cancer incidence with the most deaths among all the states in the U.S. Unfortunately, around 16% of Florida adults continue to smoke cigarettes due to its addictive nature and the limited success of current cessation strategies, partly because these cessation strategies have various adverse effects, such as suicide risk, anxiety, and insomnia. Therefore, there is an unmet and urgent need for novel interventions to improve the success of tobacco cessation. Kava is a traditional beverage consumed daily by residents of the South Pacific Islands to promote relaxation, socializing and to improve the quality of sleep. Kava has been available as a dietary supplement in the US for several decades to support calm and relaxation. Previous studies have suggested that kava supplementation may reduce tobacco use and dependence among addicted smokers. No withdraw or dependence has been observed in kava use in clinical trials or during its use as a beverage or a dietary supplement. The ultimate goal of this project is to develop a safe and effective kava-based intervention to enable tobacco cessation and reduce lung cancer risk, which will improve health. This study will use AB-free kava which is a new formulation that is expected to have a better safety profile than other kava supplements. This study will document AB-free kava use compliance, safety, evaluate reduction in tobacco dependence, and study AB-free kava’s potential benefits in reducing tobacco use and lung cancer risk.

You may be involved in this research for 12 weeks.

1. **What is involved with your participation, and what are the procedures to be followed in the research?**

The trial will enroll active smokers, who are otherwise healthy, to receive either AB-free Kava or placebo. Neither you nor your doctor will know whether you are taking AB-free Kava or placebo. Subjects will take one AB-free Kava capsule (75mg) or placebo capsule three times per day for four weeks, with two monthly follow up visits to examine longer term effects of the AB-free kava intervention and to monitor safety. In total, once enrolled, 6 visits are required over 12 weeks. Each visit while you are on the study will last 30 to 60 minutes.

During each of the of the six visits during the 4-week treatment period, you will undergo safety lab tests of your blood and urine, a Carbon Monoxide (CO) breath test, and complete questionnaires about your smoking, behaviors, addiction, urges, stress, and suicidality. An additional blood sample and 24-h urine sample (to be collected the day before the visit) will be collected for research testing. More details can be found in section 7 of this form.

1. **What are the likely risks or discomforts to you?**

Possible discomforts and risks from taking a kava supplement are minimal, specifically risk of liver toxicity, based on previous data from human studies. AB-free kava will be used in this study, which is expected to have an improved safety profile above other kava supplements. Nonetheless, any kava use may result in sedating effects such that operation of heavy instruments should be avoided and additional attention may be needed when driving within 2-hours after AB-free kava use, until you know how AB-free kava will affect you. Also built into the study is a Ask Suicide-Screening Questions form (ASQ)) to assess suicide risk given kava’s potential neurological functions, per FDA requirement, although such a risk has not been observed in studies of previous kava use. Kava use has been suggested to potentially increase the risk of liver toxicity and it is highly recommended for the participant to control alcohol use or exposure to other risk factors, such as the use of acetaminophen, during the trial period. You will be asked to refrain from taking acetaminophen while in this study and will be provided a list of medications that contain acetaminophen. You will also be asked to limit your alcohol consumption (one drink a day) while on this study. Subjects with liver conditions will be excluded and liver function will be monitored throughout the study with safety blood tests. If there are negative effects found in these tests, you will be notified immediately and retested in the next 48 – 72 hours, or completely discontinued from the study if severe and/or accompanied by other symptoms. The risks of drawing blood from a vein include discomfort at the site of puncture, possible bruising and swelling around the puncture site, rarely an infection, and, uncommonly, faintness from the procedure.

1. **What are the likely benefits to you or to others from the research?**

AB-free kava may or may not provide those randomized to the AB-free kava group a reduction in the urge to smoke and use tobacco. It may or may not also reduce damage caused by tobacco carcinogens and increase the clearance of NNAL, a known carcinogen in lung cancer, and thereby reduce lung cancer risk.

1. **What are the appropriate alternative procedures or courses of treatment, if any, that might be helpful to you?**

There are other smoking cessation programs, such as government programs available through Tobacco Free Florida, you may wish to try if you wish to stop smoking but do not wish to participate in this study. Your physician can review these with you if you wish.

A description of this clinical trial will be available on [*http://www.ClinicalTrials.gov*](http://www.ClinicalTrials.gov)*,* as required by U.S. Law.  This Web site will not include information that can identify you.  At most, the Web site will include a summary of the results. You can search this Web site at any time.

***Additional and more detailed information is provided within the remainder of this Informed Consent form, please read before deciding if you wish to participate in this study***

| What Can you Expect if you Participate in this Study? |
| --- |

6. What will be done as part of your normal clinical care (even if you did not participate in this Research Study)?

There are other smoking cessation programs, such as government programs available through Tobacco Free Florida, you may wish to try if you wish to stop smoking but do not wish to participate in this study. Your physician can review these with you if you wish.

7. What will be done only because you are in this Research Study?

You may need to have the following exams, tests or procedures to find out if you can be in the study. If you had some of these tests done recently, they may not need to be repeated. This will be up to your study doctor.

- Medical history
- Physical exam with vital signs
- Blood tests to evaluate your blood counts and blood chemistry
- Pregnancy testing (if applicable)
- Current medications review

If you are eligible and decide to take part in this study, you will be randomly assigned (much like the flip of a coin) to receive either AB-free kava or placebo. A placebo is a substance that looks like and is given in the same way as an experimental treatment but contains no medicine, for example a sugar pill. A placebo is used in research studies to show what effect a treatment has compared with taking nothing at all. If you are assigned to receive placebo, you will not receive the benefits of the AB-free kava, if there are any, nor will you be exposed to its risks, which are described below under "What are the possible discomforts and risks?" Studies have shown, however, that about 1 in 3 persons who take a placebo do improve, if only for a short time. You and the physician and other persons doing the study will not know whether you are receiving placebo or AB-free kava, but that information is available if it is needed. Also, you will have a 50% chance of receiving AB-free kava and a 50% chance of receiving placebo. In the remainder of the description of what will be done, both the AB-free kava and the placebo will be called "study treatment."

The trial will enroll active smokers, who are otherwise healthy, to be randomized into the placebo and AB-free kava arms as described above. You will take either placebo or AB-free kava for four weeks, with four visits to clinic as specified (Week 0, Week 1, Week 2, and Week 4). Your participation will involve taking one capsule three times daily at approximately 8 AM, 1PM, and 6PM. The dose of AB-free kava, if you are assigned to this group, will be 75 mg per capsule. There will be no dose modification and use will discontinue if severe side effects are detected. After this four week period, there will be 2 monthly follow up visits to monitor your health.

During each of the six visits, you will undergo safety lab tests of your blood and urine, a CO breath test, and complete questionnaires about your smoking, behaviors, addiction (i.e alcohol use), urges, stress, insomnia, and suicidality. An additional blood sample and 24-h urine sample (to collect the day before the visit) will be collected for research testing. For the urine collection, you will be given a container for collection that you will bring to your next visit. Each of these study visits should last 30 to 60 minutes.

Once this research study is completed, any information that could identify you **might** be removed from any identifiable private information or identifiable biospecimens collected. After identifiable information is removed, the information or biospecimens could be used for future research studies or distributed to another investigator for future research studies without additional informed consent from you or your legally authorized representative
If you have any questions now or at any time during this Research Study, please contact one of the Research Team members listed in question 3 of this form.

8. What identifiable health information will be collected about you and how will it be used?

The Research Team will collect demographic information, results of physical exams, blood tests, x-rays, CT scans, and other diagnostic and medical procedures, as well as medical history.

The Research Team may collect this information from other healthcare providers, such as laboratories, which are a part of this research, as well as healthcare providers that are not part of this research (other doctors, hospitals or clinics). Other professionals at the University of Florida or Shands Hospital who provide study-related care, and the University of Florida Institutional Review Board (IRB), may also collect your health information.

The Research Team listed in question 3 above will use or share your health information as described below to carry out this research study.

9. With whom will this health information be shared?

This health information may be shared with:

- The study sponsor (listed in Question 4 of this form);
- United States governmental agencies which are responsible for overseeing research, such as the Food and Drug Administration, the Department of Health and Human Services, and the Office of Human Research Protections;
- Government agencies which are responsible for overseeing public health concerns such as the Centers for Disease Control and federal, state and local health departments.
- Your insurance company for purposes of obtaining payment
- The IRB that reviewed this Research Study and ensures your rights as a Study Subject are protected

Otherwise, your identifiable health information will not be shared without your permission unless required by law or a court order. Once your health information is shared with those listed above, it is possible that they could share it without your permission because it would no longer be protected by the federal privacy law.

10. How long will you be in this Research Study?

Your participation in this study is expected to last approximately 12 weeks.

This Authorization to use and share your health information expires at the end of the study, unless you revoke it (take it back) sooner.

11. How many people are expected to take part in this Research Study?

Eighty (80) subjects are expected to be deemed eligible and take part in this study.

| What are the Risks and Benefits of this Study and  What are Your Options? |
| --- |

12. What are the possible discomforts and risks from taking part in this Research Study?

The risks of drawing blood from a vein include discomfort at the site of puncture; possible bruising and swelling around the puncture site; rarely an infection; and, uncommonly, faintness from the procedure.

Possible discomforts and risks from taking a kava supplement are minimal, based on previous data from human studies. AB-free kava will be used in the study, which is expected to have an improved safety profile above other kava supplements. Because there is a potential for AB-free kava to affect the function of the liver, subjects with liver conditions will be excluded and we will monitor liver function throughout the study with safety blood tests. You will also be asked to limit alcohol consumption and not take acetaminophen. You will be provided a list of medications containing acetaminophen. If there are negative effects found in these tests, you will be notified immediately and retested in the next 48 – 72 hours, or completely discontinued from the study if severe and/or accompanied by other symptoms. At the same time, AB-free kava use may cause sedation such that operation of heavy machinery is not recommended and additional attention is needed for driving within 2 hours after AB-free kava use, until you know how AB-free kava will affect you.

The Ask Suicide-Screening Questions form (ASQ) has been built into the study to assess the suicide risk given kava’s potential neurological functions per FDA’s suggestion, however, the risk of kava induced suicide is minimal, if any, based on previous research with no report(s) of such suicide risks in clinical trials. This is a research study that involves questions related to sensitive topics. As researchers, we do not provide mental health services. However, we want to provide you with contact information for available resources, should you decide you need assistance at any time. Here are some numbers for available resources; UF psychiatry clinical sites (352) 265-4357., the Alachua county Crisis Center (352) 264-6789, and the National Suicide Hotline 1-800-784-2433. Should an emergency arise in clinic (statement of demonstration of active suicide ideation) standard of care clinic procedures will be followed. This involves calling Alachua County Crisis Center to have an in-clinic evaluation or sending the patient directly to a psychiatric facility. Please ask the study coordinator if you would like more information on these clinic procedures.

Other possible risks to you may include emotional discomfort at responding to some survey questions. However, you are not required to answer any question you wish to skip

This Research Study may also include risks that are unknown at this time.

Please note, participating in more than one research study or project may further increase the risks to you. If you are already enrolled in a research study, please inform one of the Research Team members listed in question 3 of this form or the person reviewing this consent with you before enrolling in this or any other research study or project.

During the study, the Research Team will notify you of new information that may become available and might affect your decision to remain in the study.

The University of Florida is required by law to protect your health information. Your health information will be stored in locked filing cabinets or on computer servers with secure passwords, or encrypted electronic storage devices, as required by University policy. However, there is a slight risk that information about you could be released inappropriately or accidentally. Depending on the type of information, a release could upset or embarrass you, or possibly affect your ability to get insurance or a job.

If you wish to discuss the information above or any discomforts you may experience, please ask questions now or call one of the Research Team members listed in question 3 in this form.

13a. What are the potential benefits to you for taking part in this Research Study?

AB-free kava may or may not provide those randomized to the AB-free kava group a reduction in the urge to smoke and tobacco use. It may or may not also reduce damage caused by tobacco carcinogens and increase the clearance of NNAL, a known carcinogen in lung cancer, and thereby reduce lung cancer risk.

13b. How could others possibly benefit from this Research Study?

If AB-free kava supplementation is able to facilitate tobacco cessation and is safe, it may help inform smoking cessation programs for other healthy smokers.

13c. How could the Research Team members benefit from this Research Study?

In general, presenting research results in conferences, peer-reviewed publications helps the career of a researcher. Therefore, the Research Team listed in question 3 of this form may benefit if the results of this Research Study are presented at scientific meetings or in scientific journals. The results may also lead to new knowledge and experience that will help expand and initiate similar studies in the future.

Dr. Chengguo Xing, Co-Investigator, might benefit financially from this study. Specifically, Dr. Xing is the inventor of the new treatment being studied, and Dr. Xing has an investment in Kuality Herbceutics, such as stock. Kuality Herbceutics is a start-up company that was created to make the new treatment widely available. Research studies, like the one you are thinking of joining, are done to determine whether the new treatment is safe and effective. If research shows the new treatment is safe and effective, Dr. Xing may receive a part of the profits from any sales. In addition, the amount of money Dr. Xing’s investment is worth might be affected by the results of this study. This means that Dr. Xing could gain or lose money depending on the results of this study. The Institutional Review Board and the University of Florida have reviewed the possibility of the financial benefit. They believe that the possible financial benefit to the person leading the research is not likely to affect your safety and/or the scientific quality of the study. If you would like more information, please ask the researchers or study coordinator.

**13d. Will you be allowed to see the research information collected about you for this Research Study?**

You may not be allowed to see the research information collected about you for this Research Study, including the research information in your medical record, until after the study is completed. When this Research Study is over, you will be allowed to see any research information collected and placed in your medical record.

14. What other choices do you have if you do not want to be in this study?

You may choose not to participate in this study. There are other smoking cessation programs you may wish to try if you wish to stop smoking but do not wish to participate in this study, such as government programs available through Tobacco Free Florida. Your physician can review these with you if you wish.

You may also refuse to authorize the use of your health information, but if you refuse, you may not be allowed to be in this research study or receive any research-related treatment that is only available in this research study. However, your decision not to sign this Authorization will not affect any other treatment you may be eligible to receive.

15a. Can you withdraw from this study?

You may withdraw your consent and stop participating in this Research Study at any time. If you do withdraw your consent, there will be no penalty to you, and you will not lose any benefits to which you are otherwise entitled.

If you decide to withdraw your consent to participate in this Research Study for any reason, please contact the Research Team listed in question 3 of this form. They will tell you how to safely stop your participation.

You can also change your mind and take back this Authorization at any time by contacting the Research Team listed in question 3 of this form to let them know your decision. If you take back this Authorization, the Research Team may only use and disclose your health information already collected for this research study. No additional health information about you will be collected or disclosed to the Research Team. However, if you take back this Authorization, you may not be able to continue in this study. Please discuss this with a member of the Research Team listed in question #3.

15b. Can the Principal Investigator withdraw you from this Research Study?

You may be withdrawn from this Research Study without your consent for the following reasons:

- Your lab tests show it is not safe for you to continue, or your physician believes it is unsafe for you to continue treatment
- You are not compliant with the treatment schedule

| What are the Financial Issues if You Participate? |
| --- |

16. If you choose to take part in this Research Study, will it cost you anything?

**Study Drug**

The study drug, AB-free kava or placebo will be provided at no cost to you while you are participating in this study.

**Study Services**

No, there will be no additional costs to you or your health plan as a result of your participation in this study. The sponsor will pay for all health care costs related to your participation, including all required study items, services and procedures described in this consent form. However, if you feel you have received a bill related to this study, please contact the Principal Investigator.

If you receive any healthcare at UF Health that is not related to this study, this care will be billed as usual.

17. Will you be paid for taking part in this Research Study?

You will be paid $50 per study visit for a total of up to $300.

If you are paid more than $199 for taking part in this study, your name and social security number will be reported to the appropriate University employees for purposes of making and recording the payment as required by law. You are responsible for paying income taxes on any payments provided by the study. Payments to ***nonresident aliens*** must be processed through the University of Florida Payroll and Tax Services department. If the payments total $600 or more in a calendar year, the University must report the amount you received to the Internal Revenue Service (IRS). The IRS is not provided with the study name or its purpose. If you have questions about the collection and use of your Social Security Number, please visit: <http://privacy.ufl.edu/SSNPrivacy.html>.

Your payment for participation in this research study is handled through the University of Florida’s Research Participant Payments (RPP) Program.  Your information which will include your name, address, date of birth, and SSN (depending on amount of money you are paid) is protected.  Access to the (RPP) Program site is limited to certain staff with the assigned security role.  You will be randomly assigned a specific identification (ID) number to protect your identity.

If you have any problems regarding your payment contact the study coordinator listed in question 3.

18. What if you are injured while in this Research Study?

If you are injured as a direct result of your participation in this study, only the professional services that you receive from any University of Florida Health Science Center healthcare provider will be provided without charge. These healthcare providers include physicians, physician assistants, nurse practitioners, dentists or psychologists. Any other expenses, including Shands hospital expenses, will be billed to you or your insurance provider.

You will be responsible for any deductible, co-insurance, or co-payments. Some insurance companies may not cover costs associated with research studies or research-related injuries. Please contact your insurance company for additional information.

The Principal Investigator will determine whether your injury is related to your participation in this study.

No additional compensation is routinely offered. The Principal Investigator and others involved in this study may be University of Florida employees. As employees of the University, they are protected under state law, which limits financial recovery for negligence.

Please contact one of the research team members listed in question 3 of this form if you experience an injury or have questions about any discomforts that you experience while participating in this study.

| **Signatures** |
| --- |

As an investigator or the investigator’s representative, I have explained to the participant the purpose, the procedures, the possible benefits, and the risks of this Research Study; the alternative to being in the study; and how the participant’s protected health information will be collected, used, and shared with others:

|  |  |  |
| --- | --- | --- |
| Signature of Person Obtaining Consent and Authorization |  | Date |

You have been informed about this study’s purpose, procedures, possible benefits, and risks; the alternatives to being in the study; and how your protected health information will be collected, used and shared with others. You have received a copy of this Form. You have been given the opportunity to ask questions before you sign, and you have been told that you can ask questions at any time.

You voluntarily agree to participate in this study. You hereby authorize the collection, use and sharing of your protected health information as described above. By signing this form, you are not waiving any of your legal rights.

|  |  |  |
| --- | --- | --- |
| Signature of Person Consenting and Authorizing |  | Date |

**Appendix 2: Biological Specimens**

**Blood processing:**

Each visit:

1. Blood (10 mL) will be collected.
2. Within 10 minutes of collection, blood in the 10mL purple-top tube needs to be processed.
   - <https://www.youtube.com/watch?v=TKxJ4eYkt4M>
   - Blood will be separated into its respective components (plasma, buffy coat, and RBCs) using a centrifuge.
   - Spin the blood tubes to further define the buffy coat layer (1200g for 10 min).
   - Using a plastic pipette, withdraw plasma and place in a cryogenic storage vial (4 mL), leaving behind ~2-3 mm of the plasma above the buffy coat.
   - Using a plastic pipette, carefully aspirate the buffy coat from the tube, and transfer to a cryogenic storage vial (2 mL). Include ~2 mm of the RBC directly below the buffy coat boundary.
   - Using a plastic pipette, transfer RBCs into a cryogenic storage vial (4 mL).
3. Store all blood samples at -20o C. These will be transferred to the Xing lab to Xingor Freeman, which will be stored at -80C. The sample inventory will be updated.

Supplies needed at the clinic: 10-mL purple-top tube (to be purchased at the clinic), plastic pipette (stocked at the clinic), and cryogenic storage vial (stocked at the clinic).

**Urine collection and processing:**

Each visit:

Collection:

1. Provide participant with one 3L collection jug (Neta SIM-B350or Fisher 82028-222), female participants additional with commode specimen collection “hat” (McKesson 16-9522-CS100), a cold pack and a cool bag (<https://hotcoldbads.com>). The urine jug will be labeled with the sample following the Sample Labeling Codes.
2. Instruct participant to start the collection AFTER they first wake up and urinate for the day. They will mark the time of their first void and then collect ALL their urine for 24 hrs. (i.e. if participant wakes at 9 am, they do not collect that urine, but marks 9 am as the start time. They will then collect all urine until 9 am on the following day starting with the 2nd time they urinate on the first day).
3. Participant should return the urine to the clinic the day they complete the collection. Query as to whether any voids were missed.
4. Sample should be kept cool in the cool bag with the cold pack (i.e. not kept in hot car; the cold pack should be cooled in the freezer before use) and transferred to Xing lab.
5. Sample prep for storage (to be performed in Xing lab):

- DO NOT fill past the last gradient mark or the tubes/bottles may rupture upon freezing.
- Invert jug several times prior to aliquoting to make sure sample is well mixed.
- Measure the total urine volume in designated 500 mL glass graduated cylinder to the closest 10 mL.
- Make 5 aliquots of 3.5 mL in 4 mL cryovials (VWR –82050-218), 2 aliquotsin 50 mL tube (Fisher –14-7432-22), and one aliquot in 250 mL polypropylene bottles (VWR 414004-125).
- Store at -80^o^ C Freezer

Supplies needed at the clinic: 3-L urine collection container (stocked at the clinic), urine hats (stocked at the clinic), cool bag (stocked at the clinic), and cold pack (stocked at the clinic).
